# Killer cell proteases can target viral immediate-early proteins to control human cytomegalovirus infection in a noncytotoxic manner

**DOI:** 10.1371/journal.ppat.1008426

**Published:** 2020-04-13

**Authors:** Liling Shan, Shuang Li, Jan Meeldijk, Bernadet Blijenberg, Astrid Hendriks, Karlijn J. W. M. van Boxtel, Sara P. H. van den Berg, Ian J. Groves, Martin Potts, Adriana Svrlanska, Thomas Stamminger, Mark R. Wills, Niels Bovenschen

**Affiliations:** 1 Department of Pathology, University Medical Center Utrecht, Utrecht, The Netherlands; 2 Center for Translational Immunology, University Medical Center Utrecht, Utrecht, The Netherlands; 3 Department of Medicine, University of Cambridge, Cambridge, United Kingdom; 4 Institute of Clinical and Molecular Virology, University of Erlangen-Nuremberg, Erlangen, Germany; 5 Institute for Virology, Ulm University Medical Center, Ulm, Germany; Thomas Jefferson University, UNITED STATES

## Abstract

Human cytomegalovirus (HCMV) is the most frequent viral cause of congenital defects and can trigger devastating disease in immune-suppressed patients. Cytotoxic lymphocytes (CD8^+^ T cells and NK cells) control HCMV infection by releasing interferon-γ and five granzymes (GrA, GrB, GrH, GrK, GrM), which are believed to kill infected host cells through cleavage of intracellular death substrates. However, it has recently been demonstrated that the *in vivo* killing capacity of cytotoxic T cells is limited and multiple T cell hits are required to kill a single virus-infected cell. This raises the question whether cytotoxic lymphocytes can use granzymes to control HCMV infection in a noncytotoxic manner. Here, we demonstrate that (primary) cytotoxic lymphocytes can block HCMV dissemination independent of host cell death, and interferon-α/β/γ. Prior to killing, cytotoxic lymphocytes induce the degradation of viral immediate-early (IE) proteins IE1 and IE2 in HCMV-infected cells. Intriguingly, both IE1 and/or IE2 are directly proteolyzed by all human granzymes, with GrB and GrM being most efficient. GrB and GrM cleave IE1 after Asp^398^ and Leu^414^, respectively, likely resulting in IE1 aberrant cellular localization, IE1 instability, and functional impairment of IE1 to interfere with the JAK-STAT signaling pathway. Furthermore, GrB and GrM cleave IE2 after Asp^184^ and Leu^173^, respectively, resulting in IE2 aberrant cellular localization and functional abolishment of IE2 to transactivate the HCMV UL112 early promoter. Taken together, our data indicate that cytotoxic lymphocytes can also employ noncytotoxic ways to control HCMV infection, which may be explained by granzyme-mediated targeting of indispensable viral proteins during lytic infection.

## Author summary

Human cytomegalovirus (HCMV) is the leading viral cause of congenital defects, can trigger disease in immune-compromised patients, and plays roles in cancer development. Cytotoxic lymphocytes kill HCMV-infected cells via releasing a set of five cytotoxic serine proteases called granzymes. However, the killing capacity of cytotoxic cells is limited and multiple T cell hits are required to kill a single virus-infected cell. This raises the question whether cytotoxic lymphocytes can use granzymes to control HCMV infection in a noncytotoxic manner. Here, we show that cytotoxic lymphocytes can also use granzymes to inhibit HCMV replication in absence of cell death. All five granzymes cleave and inactivate both viral immediate-early (IE1/2) proteins, which are essential players for initiating HCMV infection. Our data support the model that cytotoxic cells employ granzymes to dampen HCMV replication prior to accumulation of sufficient hits to kill the infected cell.

## Introduction

Human cytomegalovirus (HCMV) is a member of the beta-*herpesviridae* family with worldwide seroprevalence of up to 90% [1]. It is the most frequent viral cause of congenital defects and HCMV may promote tumor development [1, 2]. Primary infection induces a life-long latent infection, in bone marrow-resident precursor cells of the myeloid lineage (CD34^+^ hematopoietic progenitor cells), amongst others [3]. Differentiation of these latently infected myeloid precursors into migrating macrophages or mature dendritic cells is the proposed mechanism for viral organ dissemination and reactivation from latency [3]. HCMV replication is normally controlled by a vigorous host immune response [1]. However, in the absence of an adequate immune response, *e*.*g*. following immune suppression after allogeneic stem cell transplantation (SCT), solid organ transplantation, or in untreated HIV patients, HCMV can cause invasive diseases with end-organ failure, morbidity, and mortality [1]. No effective vaccine is available and anti-viral drugs are limited due to toxicity and emergence of drug-resistant virus [4, 5].

The HCMV virion consists of an icosahedral nucleocapsid containing a 230-kbp DNA genome that is surrounded by a lipid bilayer. In between the capsid and lipid bilayer is a protein-rich space called the tegument, which contains proteins that facilitate HCMV replication [1, 6, 7]. Activation of the HCMV major immediate-early (IE) promoter leads to expression of IE1 and IE2 proteins, which are silenced during the latent state and essential for HCMV reactivation and production of infectious virus [3, 6]. During productive infection, IE1 and IE2 are key to trigger a temporally coordinated cascade of transcriptional events that lead to expression of early (E) and late (L) viral proteins [6, 8].

Cytotoxic lymphocytes (*e*.*g*. antigen-specific CD8^+^ T cells and NK cells) are critical effector cells in controlling HCMV infection [9, 10]. These cytotoxic lymphocytes produce the cytokine interferon-γ (IFN-γ) to block HCMV replication [11, 12]. In addition, they release cytotoxic granules towards infected host cells via the granule-exocytosis pathway [9, 12, 13]. These granules contain the pore-forming protein perforin (PFN) and a family of five structurally homologous serine proteases called granzymes (GrA, GrB, GrH, GrK, GrM) that display distinct proteolytic substrate specificities [14, 15]. While PFN facilitates the entry of granzymes into infected cells, granzymes are believed to be the death executors during the antiviral immune effector response through cleavage of intracellular death substrates [15–17].

Recently, however, it has been demonstrated by intravital imaging that the *in vivo* killing capacity of cytotoxic T cells is limited in that multiple hits by T cells are needed to kill a single CMV-infected cell [18]. This raises the question whether cytotoxic lymphocytes can use granzymes to control HCMV infection in a noncytotoxic manner. In the present study, we demonstrate that (donor-derived HCMV-specific) CD8^+^ T cells and NK cells can inhibit HCMV replication in the absence of host cell death and independent of IFN-α/β/γ. Prior to killing, cytotoxic lymphocytes induce the degradation of IE proteins in HCMV-infected cells. We also show that all human granzymes can directly target and cleave viral IE1 and/or IE2, likely to inactivate their function and subsequent HCMV replication. Thus, besides inducing apoptosis, cytotoxic lymphocytes can also utilize noncytotoxic ways to control HCMV infection, which may be explained by granzyme-mediated targeting of indispensable viral proteins during the earliest phase of the HCMV replication cycle.

## Results

### Cytotoxic lymphocytes can inhibit HCMV dissemination in a noncytotoxic manner and can induce IE degradation in infected cells

**Fig 1:**
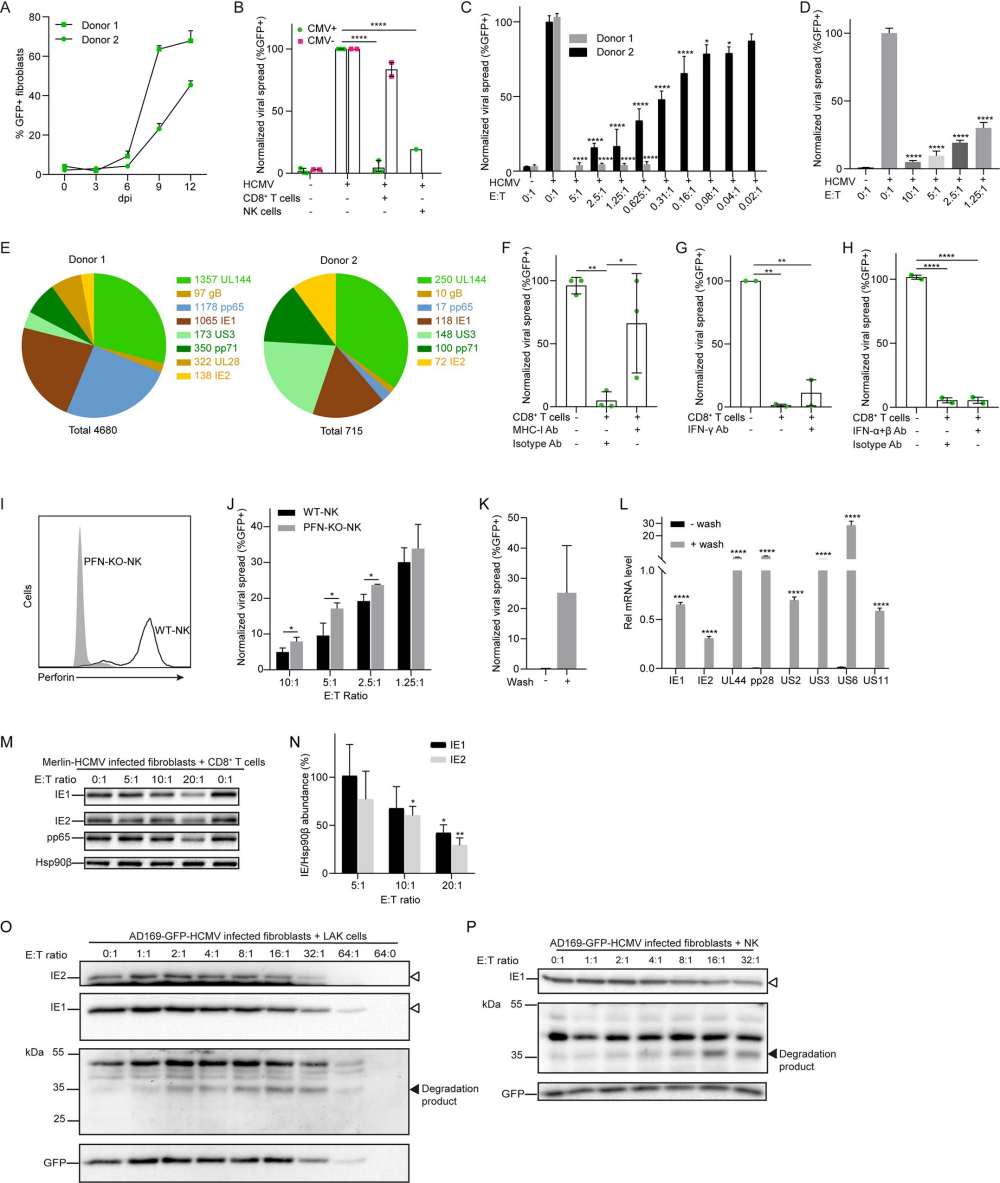
Cytotoxic lymphocytes can inhibit HCMV dissemination in a noncytotoxic manner and can induce IE degradation in infected cells. (A) CMV+ donor-derived fibroblasts were infected with GFP-HCMV (MOI = 0.05) and harvested at indicated time points for GFP quantification by flow cytometry (data represent the mean ± SD, each performed in triplicate). (B) Fibroblasts were infected with GFP-HCMV (MOI = 0.08) and co-cultured with autologous CD8^+^ T cells (E:T = 2.5:1) from CMV+ donors (data represent the mean with range of 3 donors, each performed in triplicate), CMV- donors (data represent the mean with range of 2 donors, each performed in triplicate), or non-autologous NK cells (E:T = 2.5:1) for 9–14 days. (C) GFP-HCMV (MOI = 0.08) infected CMV+ donor-derived fibroblasts were co-cultured with autologous CD8^+^ T cells at indicated E:T ratios for 9 days (data represent the mean ± SD, each performed in triplicate). (D) GFP-HCMV (MOI = 0.08) infected CMV+ donor-derived fibroblasts were co-cultured with non-autologous NK cells at indicated E:T ratios for 9 days (data represent the mean ± SD, n = 3). (E) Absolute numbers (IFN-γ Spot Forming Units SFU/10^6^ cells) of HCMV-specific CD8^+^ T cells (stimulated with overlapping peptide pools UL144, gB, pp65, IE1, IE2, US3, UL28, pp71) derived from two CMV+ donors as determined by FluoroSpot IFN-γ assay. (F) Fibroblasts were infected with GFP-HCMV (MOI = 0.08) and co-cultured with autologous CD8^+^ T cells (E:T = 2.5:1) in the absence or presence of MHC-I blocking antibody (data represent the mean ± SD of 3 CMV+ donors, each performed in triplicate). (G) Fibroblasts were infected with GFP-HCMV (MOI = 0.08) and co-cultured with autologous CD8^+^ T cells (E:T = 2.5:1) in the absence or presence of IFN-γ blocking antibody (data represent the mean with range of 2 CMV+ donors, each performed in triplicate). (H) Fibroblasts were infected with GFP-HCMV (MOI = 0.08) and co-cultured with autologous CD8^+^ T cells (E:T = 2.5:1) in the absence or presence of IFN-α and IFN-β blocking antibodies (data represent the mean with range of 2 CMV+ donors, each performed in triplicate). (I) FACS histogram visualizing PFN protein levels in WT-NK cells and PFN-knockout (KO) NK cells. (J) GFP-HCMV (MOI = 0.08) infected CMV+ donor-derived fibroblasts were co-cultured with non-autologous WT or PFN-KO NK cells at indicated E:T ratios for 9 days (data represent the mean ± SD, n = 3). (K) Fibroblasts were infected with GFP-HCMV (MOI = 0.08) and co-cultured with autologous CD8^+^ T cells (E:T = 2.5:1) of one CMV+ donor for 10 days. Then, CD8^+^ T cells were either left or washed out and fibroblasts were cultured for another 10 days. GFP+ cells were measured (data represent the mean ± SD, n = 3). (L) Fibroblasts were infected with GFP-HCMV (MOI = 0.08) and co-cultured with autologous CD8^+^ T cells (E:T = 2.5:1) of one CMV+ donor for 10 days. Then, CD8^+^ T cells were either left or washed out and fibroblasts were cultured for another 10 days. Viral mRNA was measured (data represent the mean ± SD, n = 3). (M) Fibroblasts were infected with Merlin delta US2-11-HCMV (MOI = 0.8) and treated with autologous IE1-specific CD8^+^ T cells (from one donor) at indicated E:T ratios for 24 h, followed by immunoblotting using antibodies against IE1/2, pp65 and Hsp90β. (N) IE1/2 and Hsp90β band intensities (see M) were semi-quantified. IE/Hsp90β ratio in absence of CD8^+^ T cells was set to 100% (data represent the mean ± SD, n = 3). (O) HFFs were infected with GFP-HCMV (MOI = 0.4) and treated with LAK cells at indicated E:T ratios for 6 h, followed by immunoblotting using antibodies against IE1/2 and GFP. (P) HFFs were infected with GFP-HCMV (MOI = 0.4) and treated with NK cells at indicated E:T ratios for 6 h, followed by immunoblotting using antibodies against IE1 and GFP. White and black triangles indicate full length IE proteins and IE degradation products, respectively. Statistical analysis was performed using one-way ANOVA with Turkeys multiple comparisons test (B, F, G, H) or Student t test (C, D, J, K, L, N). *P<0.05, **P<0.01, ****P<0.0001.

Recently, it has been demonstrated by intravital imaging that the *in vivo* killing capacity of cytotoxic T cells is limited, in that multiple T cell hits are required to kill a single CMV-infected cell [18]. This raises the question whether cytotoxic cells can also control HCMV infection in a noncytotoxic manner. To address this hypothesis, a viral dissemination assay was developed to monitor viral replication and spread. Donor-derived fibroblasts were infected with GFP-HCMV (Merlin) at low multiplicity of infection (MOI) and the percentage of HCMV-infected fibroblasts increased over a time course as measured by flow cytometry, indicating viral replication and subsequent spread to uninfected cells as would be expected (Fig 1A). Next, we co-cultured low MOI GFP-HCMV-infected fibroblasts with autologous CD8^+^ T cells or non-autologous NK cells. Low MOI infected fibroblasts with no co-culture of lymphocytes was also included as an infection control. The cultures were incubated for 9–14 days to allow multiple rounds of viral infection and spread through the culture. Following incubation, the fibroblasts were examined for GFP expression by flow cytometry. As expected [19], CD8^+^ T cells from HCMV+ donors, but not from HCMV- donors, and NK cells could control viral replication and spread (Fig 1B). Both CD8^+^ T cells (Fig 1C) and NK cells (Fig 1D) control HCMV in an effector:target (E:T) cell-dependent manner, and the efficiency of HCMV inhibition positively correlated to the frequency of HCMV-specific CD8^+^ T cells in the donor as determined by overlapping peptide pools of immunodominant HCMV ORFs in a quantitative IFN-γ assay (Fig 1C and 1E). The HCMV controlling effect of autologous CD8^+^ T cells was antagonized by an anti-MHC-I antibody, confirming that antigen presentation via MHC-I and T cell recognition by CD8^+^ T cells was required to block HCMV fibroblast dissemination (Fig 1F). A neutralizing anti-IFN-γ blocking antibody only minimally restored viral spread (Fig 1G) and no neutralizing effects were observed with anti-IFN-α and IFN-β neutralizing antibodies (Fig 1H), indicating that CD8^+^ T cells also control HCMV by IFN-α/β/γ-independent pathways. In addition, PFN was knocked-out in an NK cell line by CRISPR/Cas (Fig 1I) and PFN-KO-NK cells less efficiently controlled viral spread as compared to WT-NK cells in a co-culture viral dissemination assay (Fig 1J), conforming the role of the granule exocytosis pathway in controlling HCMV. Intriguingly, HCMV viral spread (Fig 1K) and viral mRNA expression (Fig 1L) were restored following removal of CD8^+^ T cells from the co-culture, suggesting that cytotoxic cells can target HCMV replication in a noncytotoxic manner.

Next, we investigated the effects of cytotoxic cells on IE proteins in HCMV-infected fibroblasts. Viral IE1 and IE2, and downstream viral pp65, protein abundance in host cells decreased with increasing E:T ratio of autologous IE1-specific CD8^+^ T cells (Fig 1M). In contrast, cellular protein Hsp90β remained stable at different E:T ratios, further indicating that CD8^+^ T cells can target IE proteins in the absence of host cell death. After correction for Hsp90β, the relative IE1 and IE2 protein abundance decreased with increasing amounts of CD8^+^ T cells (Fig 1N). Similar results were observed when GFP-HCMV infected fibroblasts were challenged with either primary lymphokine activated killer (LAK) (Fig 1O) or NK cells (Fig 1P). GFP was used as loading control and as an indicator of host cell death in these experiments. Although GFP decreased at high E:T ratios (>16:1) likely due to cell death induced by LAK cells, the reduction of IE1 and IE2 protein abundance already occurred at low E:T ratios (>2:1). Intriguingly, an IE protein degradation product (~35 kDa) appeared at an E:T ratio of 1:1 that accumulated with increasing E:T ratios (Fig 1O). IE1 protein abundance also decreased with concomitant appearance of the 35 kDa IE degradation product without any loss of GFP when GFP-HCMV infected fibroblasts were co-cultured with NK cells (Fig 1P). Collectively, these data indicate that cytotoxic lymphocytes can control HCMV replication and IE protein abundance in a noncytotoxic manner.

### Immune effector cells target IE1 and IE2

**Fig 2:**
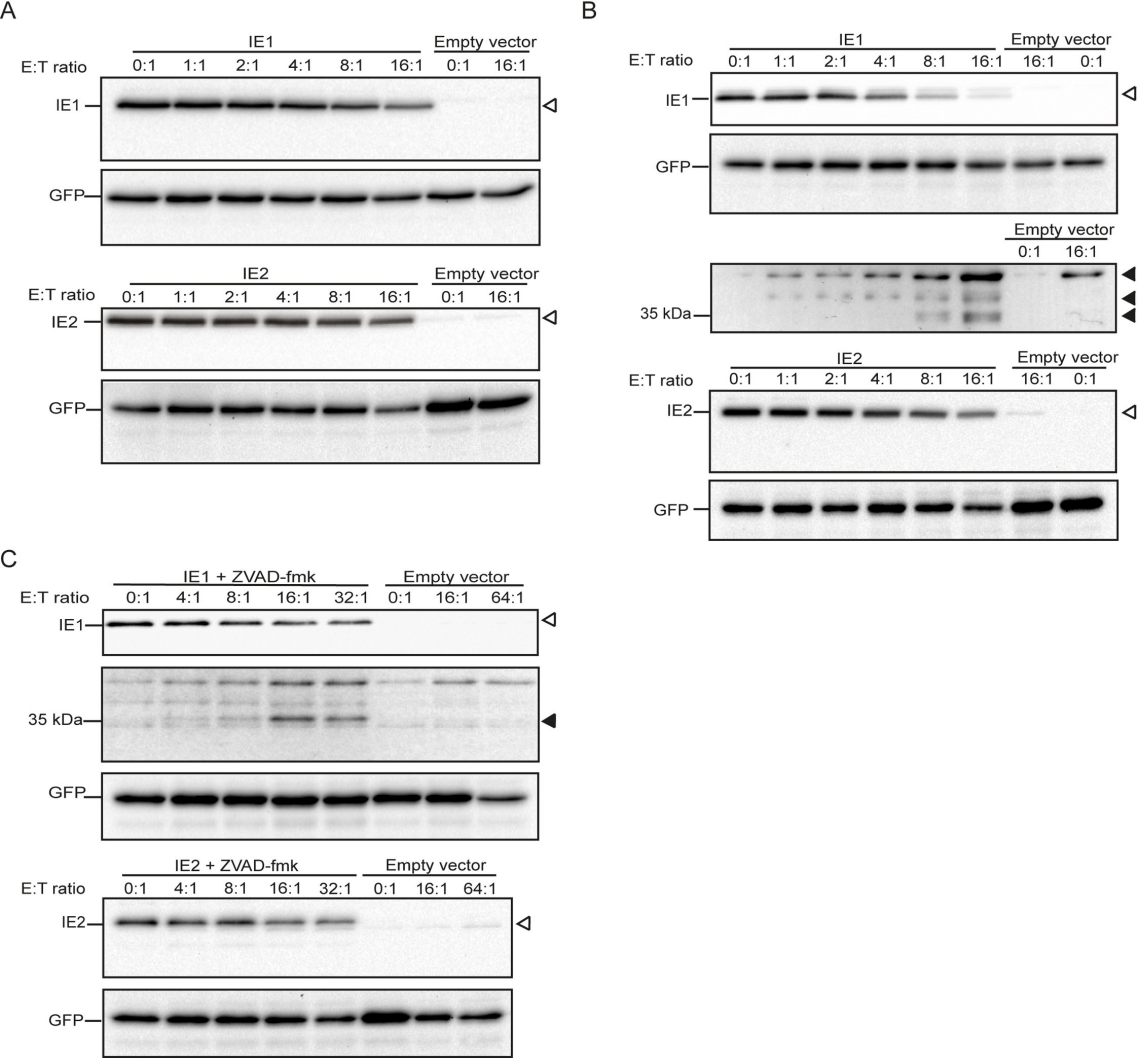
Immune effector cells target IE1 and IE2. HeLa cells were transfected with N-terminal HA-tagged IE1, IE2, or empty vector combined with GFP. 20 h post-transfection, (A) LAK cells or (B) NK cells (YT-Indy) were added with increasing E:T ratios for 6 h. (C) Caspase inhibitor ZVAD-fmk was added (100 μM) before challenging by NK cells. Lysates were subjected to immunoblotting using either an anti-HA or anti-GFP antibody. White and black triangles indicate full length IE proteins and IE degradation products, respectively.

To further investigate degradation of IE1/2 by cytotoxic cells, HeLa cells ectopically co-expressing IE1 or IE2 and GFP were co-cultured with LAK cells (Fig 2A) or NK cells (Fig 2B) for 6 h after which lysates were analyzed for IE1/2 expression by immunoblotting. GFP was used as marker for cell death (loading control). IE1 and IE2 decreased more dramatically with increasing E:T ratios than GFP (Fig 2A and 2B), and three IE1 degradation products (~55, ~45, and ~35 kDa) were detected when incubated with NK cells (Fig 2B). No degradation fragment was detected for IE2 in any of the conditions. Next, the caspase inhibitor ZVAD-fmk was added prior to addition of NK cells to inhibit cell death. The decrease in abundance of IE1/2 was still observed without loss of (cell death marker) GFP until at least the 16:1 E:T ratio (Fig 2C). In addition, the appearance of degradation fragments of IE1 was still observed in the presence of ZVAD-fmk (Fig 2C). These data indicate that immune effector cells induce degradation of IE1 and IE2 proteins, independent of cell death.

### All granzymes directly cleave HCMV IE1 and/or IE2

**Fig 3:**
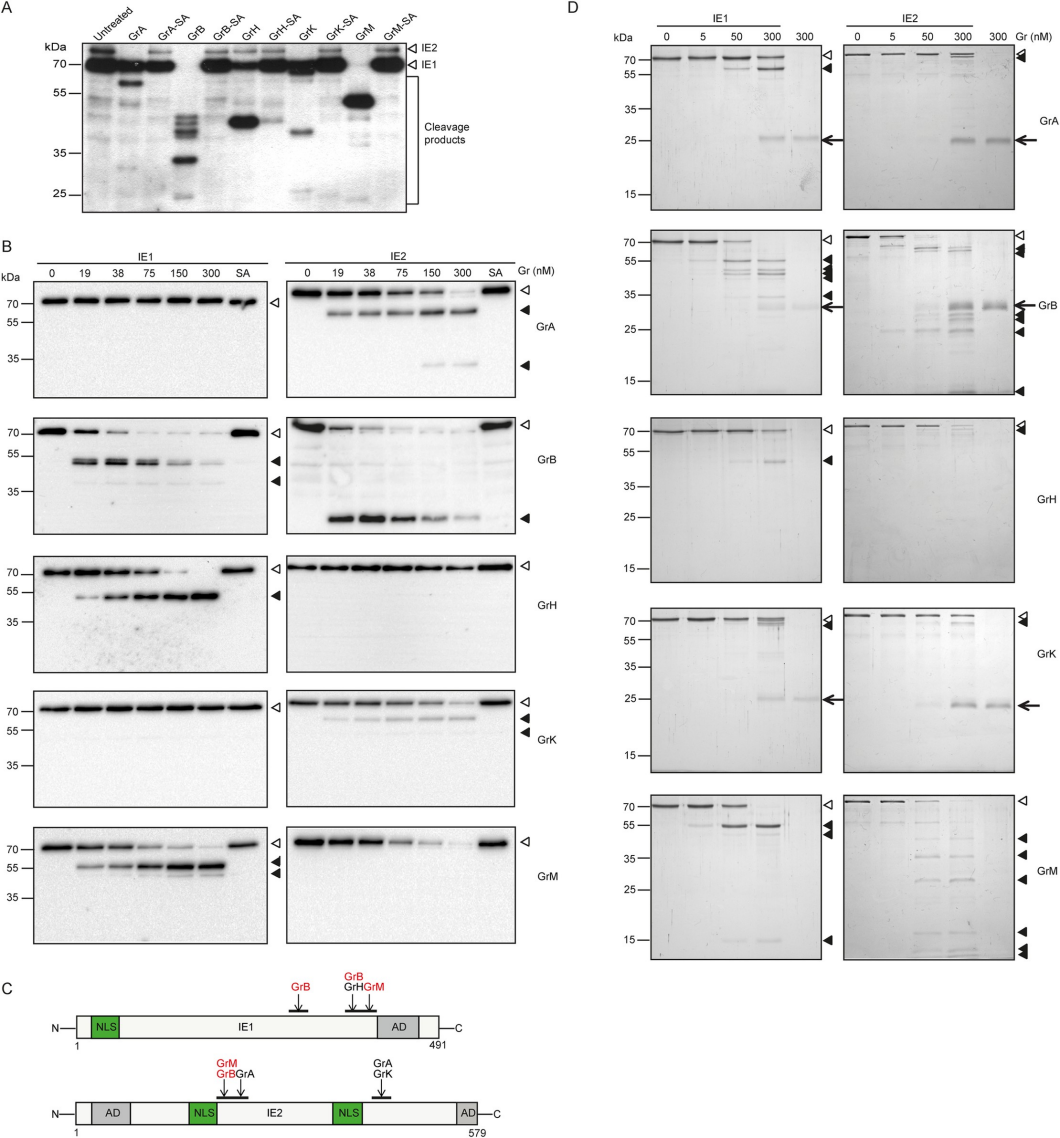
All granzymes directly cleave HCMV IE1 and/or IE2. (A) HFFs were infected with HCMV (AD169) at a MOI of 1.0. 3 days *p*.*i*., nuclear and cytosolic fractions were generated and protein concentrations were measured. Nuclear fractions (0.5 μg) were incubated with 300 nM purified human granzymes or their corresponding catalytically inactive control SA mutants, or left untreated for 4 h at 37°C and immunoblotted using an antibody against IE. (B) Cell-free lysates of HA-IE1/2 transfected HeLa cells were incubated with increasing concentrations of purified human granzymes or SA mutants (300 nM) for 3 h at 37°C and subjected to immunoblotting with an antibody against HA. (C) Schematic overview of putative human granzyme cleavage sites in IE1 and IE2 (NLS, nuclear localization signal; AD, activation domain). (D) SDS-PAGE and full protein staining of purified IE1 and IE2 incubated with five human granzymes at concentrations ranging from 0–300 nM for 3 h at 37°C. White and black triangles indicate full length IE proteins and IE cleavage products, respectively. The arrow indicates the granzymes.

Besides viral control by IFN-γ, HCMV can be controlled by the granule exocytosis pathway, in which cytotoxic cells deliver a set of granzymes (*i*.*e*., serine proteases) into virus-infected cells [12, 20]. To investigate whether the noncytotoxic degradation of HCMV IE1/2 by killer cells is the result of granzyme-mediated proteolysis, lysates of HCMV-infected Human foreskin fibroblasts (HFFs) were incubated with individual purified human granzymes or their corresponding catalytically inactive control [mutation of Ser to Ala (SA) in the catalytic center of the granzyme] after which immunoblotting was used for detection of IE cleavage. IE1 was cleaved by GrB, GrH, and GrM, whereas IE2 was cleaved by GrA, GrB, GrK, and GrM with the concomitant appearance of cleavage products (Fig 3A). To further verify the cleavage of IE1 and IE2 by human granzymes, IE1 and IE2 proteins were expressed in HeLa cells and cell-free protein lysates were incubated with increasing concentrations of individual granzymes. Again, IE1 was cleaved by GrB, GrH, and GrM, and IE2 was cleaved by GrA, GrB, GrK, and GrM in a concentration dependent manner (Fig 3B). In these experiments, all the inactive granzyme SA mutants were unable to cleave IE proteins, indicating that the catalytic serine protease activity of all granzymes is required (Fig 3A and 3B). Based on the size of N-terminal cleavage products as shown in Fig 3B, granzyme cleavage sites in IE1 and IE2 were estimated and appeared to locate in ‘hotspot’ regions (Fig 3C). Finally, to investigate whether IE1 and IE2 are direct substrates for human granzymes, purified recombinant IE1 and IE2 were incubated with increasing concentrations of individual purified granzymes and subjected to SDS-PAGE. Both IE1 and IE2 were cleaved by all granzymes with appearance of cleavage products (Fig 3D). The cleavage of IE1 and IE2 by GrB and GrM was most efficient. Taken together, these data show that all granzymes directly cleave IE1 and/or IE2 proteins.

### GrB and GrM may inhibit IE1 function

**Fig 4:**
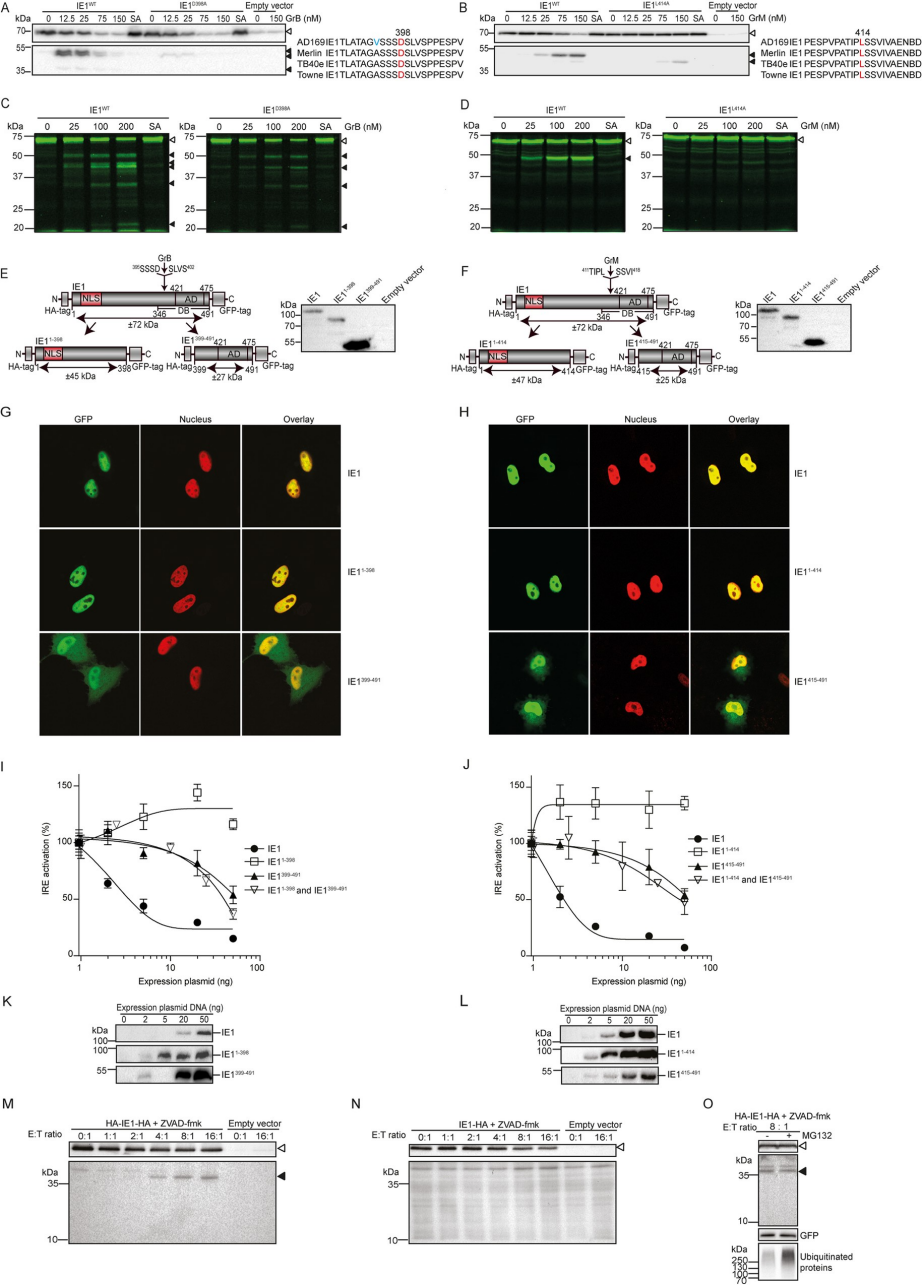
GrB and GrM may inhibit IE1 function. (A, B) HeLa cells were transfected with HA-IE1^WT^, IE1^D398A^ or IE1^L414A^ and empty vector for 24 h. Cell-free lysates were incubated with increasing concentrations of GrB (A, left panel) or GrM (B, left panel) or SA mutant (150 nM) at 37°C for 3 h and immunoblotted using an anti-HA antibody. Sequence alignment of the amino acid regions surrounding IE1^D398^ and IE1^L414^ from different HCMV strains (A and B, right panel). Amino acid in red is the P1 cleavage site and different amino acids among sequences are shown in blue. (C, D) IE1^WT^, IE1^D398A^ (C) or IE1^L414A^ (D) were labeled with fluorescent green lysines during *in vitro* transcription/translation and incubated at 37°C for 3 h with increasing concentrations of GrB (C) or GrM (D) or SA mutants (200 nM). Samples were separated by SDS-PAGE. (E, F) Left panel: Schematic overview of IE1, IE1^1-398^, and IE1^399-491^ (E), IE1, IE1^1-414^, and IE1^415-491^ (F) constructs. NLS, nuclear localization signal; AD, acidic domain; DB, DNA-binding domain. Right panel: IE1 variants were expressed in HeLa cells and lysates were subjected to immunoblotting, using an anti-HA antibody. (G, H) Immunofluorescence images of HeLa cells transfected with HA and GFP-tagged IE1, IE1^1-398^ and IE1^399-491^ (G), HA and GFP-tagged IE1, IE1^1-414^ and IE1^415-491^ (H) constructs. GFP-tagged proteins were visualized in green and nuclei were stained by co-transfection with H2B-mCherry in red. (I, J) HEK293T cells were co-transfected with pGL4.74 hRL-TK (20 ng), pGL4.24 10×IRE (100 ng), H2B-mCherry (30 ng) and increasing amounts of expression plasmids of IE1, IE1^1-398^, or/and IE1^399-491^ (I), IE1, IE1^1-414^, or/and IE1^415-491^ (J). All conditions were performed in triplicates. 24 h post-transfection, cells were incubated with 5 ng/ml IFN-β for 6 h, after which dual luciferase reporter assay was used to assess luciferase activity. Relative luciferase activity, *i*.*e*. Firefly/Renilla are depicted and the values in the absence of IE (fragments) were set to 100% of IRE activation. Bars represent the mean ± SD of three independent experiments. (K, L) lysates used in the luciferase reporter assay were subjected to immunoblotting using an anti-HA antibody. (M, N) HeLa cells were transfected with either both N- and C-terminal HA-tagged IE1 (M) or only C-terminal HA-tagged IE1 (N), complemented with H2B-GFP. After 24 h transfection, NK (YT-Indy) cells were added at indicated E:T ratios for 6 h incubation in the presence of ZVAD-fmk. Lysates were subjected to immunoblotting, using antibodies against HA and GFP. (O) HeLa cells were transfected with N- and C-terminal HA-tagged IE1 and H2B-GFP. 24 h post-transfection, cells were treated with or without MG132 (250 nM) for 30 min before challenging with NK (YT-Indy) cells at 8:1 ratio in the presence of ZVAD-fmk. Lysates were subjected to immunoblotting, using antibodies against HA, GFP, and ubiquitin. White and black triangles indicate full length IE1 proteins and IE1 cleavage products, respectively. All immunoblots are representative of at least three separate experiments.

Next, we focused on IE cleavage by GrB and GrM, since these granzymes not only cleave both IE1 and IE2 but also do this with the highest efficiency of all the granzymes (Fig 3A, 3B and 3D). To investigate the functional consequences of granzyme-mediated cleavage of IE1, the GrB and GrM cleavage sites in IE1 were estimated based on primary (P1) substrate preference [21, 22] and the molecular weight of the observed cleavage fragments (Fig 3B). Next, we constructed and expressed multiple IE1 point mutations (alanine substitutions) that allowed us to confirm Asp^398^ and Leu^414^ as GrB and GrM cleavage sites in IE1, respectively (Fig 4A–4D). Hemagglutinin (HA) -tagged IE1^WT^, IE1^D398A^, and IE1^L414A^ were expressed in HeLa cells and cell-free protein lysates were incubated with increasing GrB or GrM concentrations and analyzed by immunoblotting with an anti-HA antibody. As expected, IE1^WT^ decreased with the concomitant appearance of three N-terminal IE1 cleavage products for GrB (Fig 4A) and two IE1 cleavage products for GrM (Fig 4B). Although IE1^D398A^ abundance still decreased following GrB treatment, only one faint cleavage product remained detectable (Fig 4A). Following GrM treatment, no decrease of the IE1^L414A^ mutant was detected and the main cleavage fragment disappeared (Fig 4B). To further validate these results, we also synthesized fluorescent IE1^WT^, IE1^D398A^, and IE1^L414A^ mutants by cell-free *in vitro* transcription/translation and subsequently incubated these mutants with increasing concentrations of GrB or GrM. Consistent with immunoblotting data (Fig 4A and 4B), visualization of fluorescently labeled IE1^WT^ was cleaved by GrB and GrM with concomitant appearance of multiple cleavage fragments (Fig 4C and 4D). Treatment of IE1^D398A^ and IE1^L414A^ with GrB and GrM showed virtual undetectable IE1 cleavage fragments at 40 kDa and 45 kDa, respectively. Sequence alignment of different HCMV strains shows that the amino acid regions surrounding IE1^D398^ and IE1^L414^ are highly conserved (Fig 4A and 4B, right panel). To investigate whether GrB or GrM-cleaved IE1 is functionally active, IE1^1-398^ and IE1^399-491^ fragments that mimic GrB-cleaved IE1, and IE1^1-414^ and IE1^415-491^ fragments that mimic GrM-cleaved IE1 were generated and expressed (Fig 4E and 4F). To examine the localization, HeLa cells were transfected with the various IE1 expression plasmids. As expected [23], full length IE1 localized in the nucleus (Fig 4G and 4H, upper row). IE1^1-398^ and IE1^1-414^ still localized in the nucleus (Fig 4G and 4H, middle row), whereas cytoplasmic localization was observed for IE1^399-491^ and IE1^415-491^ (Fig 4G and 4H, bottom row). A major function of IE1 is to block the JAK-STAT signaling pathway by binding to STAT2, causing dysfunction of STAT2 as a transcription factor to initiate the Interferon Responsive Elements (IRE) [24]. As expected [24], IE1 full length efficiently inhibited cellular IRE activation following interferon-beta (IFN-β, Peprotech) treatment, as measured in a dual luciferase reporter assay (Fig 4I and 4J). In sharp contrast, IE1^1-398^ and IE1^1-414^ did not inhibit IRE activation anymore, while IE1^399-491^, IE1^415-491^, and relevant combinations still inhibited IRE activation but in a markedly less efficient manner compared to IE1 full length (Fig 4I and 4J). Immunoblot analysis showed that IE1 and its fragments were expressed (Fig 4K and 4L). Since the C-terminal IE1 cleavage fragments still slightly inhibited IRE activation, we next investigated the stability of IE1 cleavage fragments following killer cell-induced proteolysis of full length IE1 in target cells. To this end, we used IE1 variants with HA tags at the N- and/or C-terminus to discriminate between N- and C-terminal IE1 cleavage fragments. Following incubation with NK cells, one IE1 N-terminal cleavage fragment (~ 35 kDa) was detected (Fig 4M), whereas no IE1 C-terminal fragment was seen (Fig 4M and 4N). Addition of proteasome inhibitor MG132 did not rescue IE1 C-terminal fragments, while ubiquitinated proteins were rescued (positive control) (Fig 4O). These data suggest that IE1 C-terminal fragments are unstable but not degraded by the proteasome. Collectively, these data show that GrB and GrM cleave IE1 at least after Asp^398^ and Leu^414^, respectively, likely resulting in IE1 aberrant cellular localization, IE instability, and functional impairment of IE1 to interfere with the JAK-STAT signaling pathway.

### GrB and GrM abolish IE2 function

**Fig 5:**
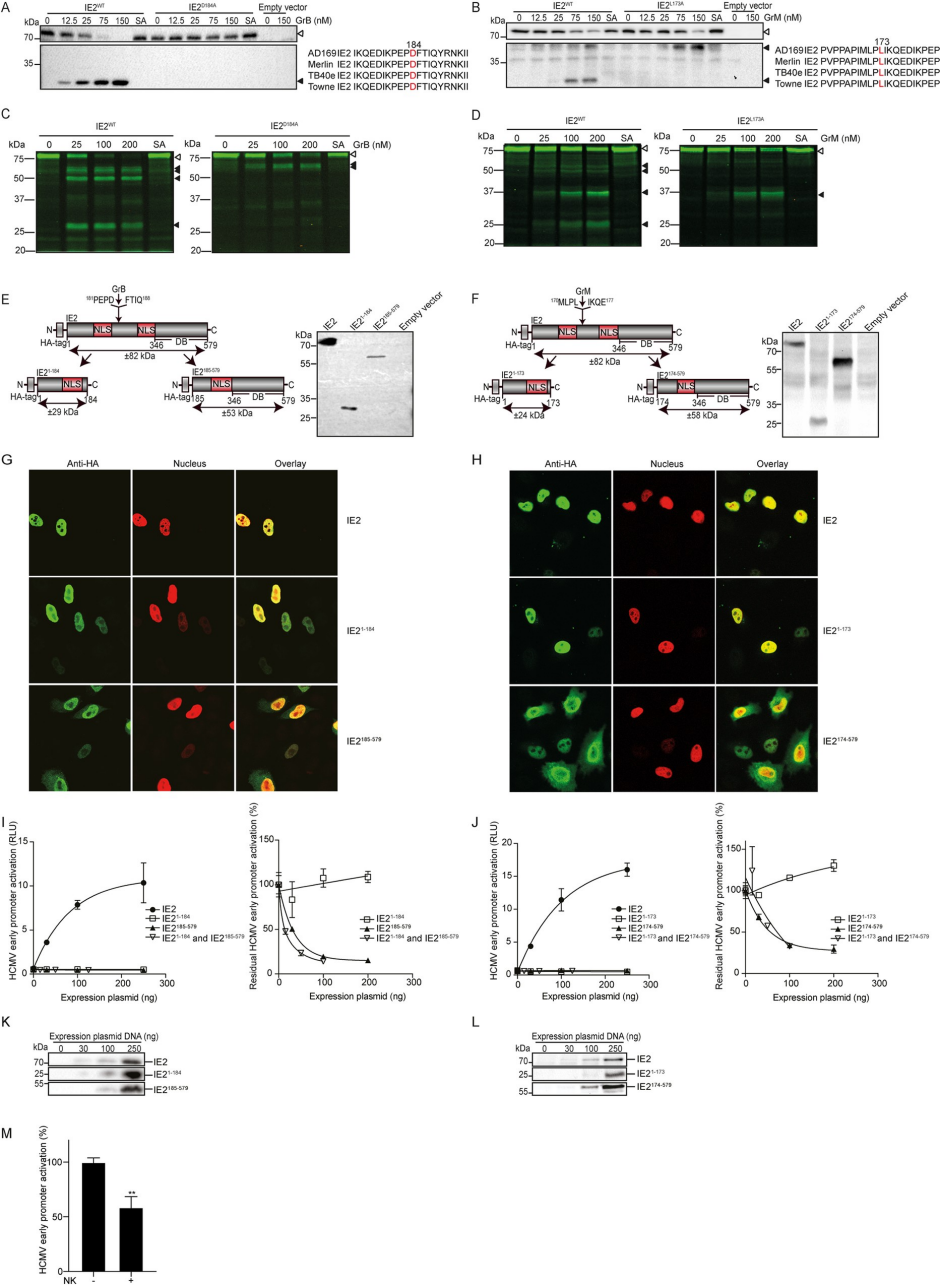
GrB and GrM abolish IE2 function. (A, B) HeLa cells were transfected with HA-IE2^WT^, IE2^D184A^, or IE2^L173A^, or empty vector for 24 h. Cell-free lysates were incubated with increasing concentrations of GrB (A, left panel) or GrM (B, left panel) or SA (150 nM) at 37°C for 3 h. Lysates were subjected to immunoblotting, using an anti-HA antibody. Sequence alignment of the amino acid regions surrounding IE2^D184^ and IE2^L173^ from different HCMV strains (A and B, right panel). Amino acids in red represent P1 cleavage sites. (C, D) IE2^WT^, IE2^D184A^ (C) or IE2^L173A^ (D) were labeled with fluorescent green lysines during *in vitro* transcription/translation and incubated at 37°C for 3 h with increasing concentrations of GrB (C) or GrM (D) or SA (200 nM). Samples were separated by SDS-PAGE. (E, F) Left panel: Schematic overview of the IE2, IE2^1-184^, and IE2^185-579^ (E), IE2, IE2^1-173^, and IE2^174-579^ (F) constructs. NLS, nuclear localization signal; DB, DNA-binding domain. Right panel: IE2 variants were expressed in HeLa cells and lysates were subjected to immunoblotting, using an anti-HA antibody. (G, H) Immunofluorescence images of HeLa cells transfected with HA-tagged IE2, IE2^1-184^, and IE2^185-579^ (G), HA-tagged IE2, IE2^1-173^, and IE2^174-579^ (H) constructs. HA-tagged proteins were visualized in green and nuclei were stained by co-transfection with H2B-mCherry in red. (I, J) HeLa cells were co-transfected with pGL4.74 TK-hRL (20 ng) (Renilla control), pGL4.11-EP Luc2 (100 ng) (UL112 Early Promoter, EP), H2B-mCherry (30 ng) and increasing amounts of IE2, IE2^1-184^, and IE2^185-579^ (I, left panel), or IE2, IE2^1-173^, and IE2^174-579^ (J, left panel). All conditions were performed in triplicate. Dual luciferase reporter assay was used to measure UL112 early promoter activation after 24 h transfection. pcDNA3.1-IE2 (50 ng), H2B-mCherry (30 ng), pGL4.74 hRL-TK (20 ng), pGL4.11-EP (100 ng), increasing amounts of pcDNA3.1-IE2^1-184^ or/and pcDNA3.1-IE2^185-579^ (I, right panel), or pcDNA3.1-IE2^1-173^ or/and pcDNA3.1-IE2^174-579^ (J, right panel), complemented with empty vector up to 400 ng total DNA were transfected in HeLa cells. All conditions were performed in triplicate. After 24 h transfection, dual luciferase reporter assay was used to measure early promoter activation. Relative luciferase activity, *i*.*e*. Firefly/Renilla, is depicted and the values from conditions in absence of IE were set to 100% early promoter activation. Bars represent the mean ± SD of three independent experiments. (K, L) Lysates used in the luciferase reporter assay were subjected to immunoblotting, using an anti-HA antibody. White and black triangles indicate full length IE2 proteins and IE2 cleavage products, respectively. All immunoblots are representative of at least three separate experiments. (M) HEK293T cells were co-transfected with H2B-mCherry (30 ng), pGL4.74 hRL-TK (20 ng), pGL4.11-EP (UL112 Early Promoter) (100 ng), with or without pcDNA3.1-HA-IE2 (10 ng), complemented with empty vector to a total of 400 ng DNA per well. After 24 h transfection, cells were cultured with or without NK cells (YT-Indy) for 23 h. Dual luciferase reporter assay was used to measure UL112 early promoter activation. Corrected relative Firefly/Renilla ratios in the absence of NK cells were set to 100%. Data represent the mean ± SD, n = 3. Student t test was used for statistical analysis, **P<0.01.

To investigate the functional consequences of granzyme-mediated cleavage of IE2, multiple IE2 point mutants (alanine substitutions) were constructed and expressed that allowed us to identify Asp^184^ and Leu^173^ as GrB and GrM-cleavage sites in IE2, respectively (Fig 5A–5D). HA-tagged IE2^WT^, IE2^D184A^, and IE2^L173A^ were expressed in HeLa cells and cell-free lysates were incubated with increasing GrB or GrM concentrations and subjected to immunoblotting with an anti-HA antibody. As expected, IE2^WT^ abundance decreased with concomitant appearance of one N-terminal cleavage product for GrB (Fig 5A) and two cleavage products for GrM (Fig 5B). No decrease of IE2^D184A^ abundance and no cleavage product were detected following GrB treatment (Fig 5A). IE2^L173A^ abundance still decreased following GrM treatment, but less efficient as compared with IE2^WT^, and only one cleavage product remained detectable (Fig 5B). To further validate these results, fluorescently labeled IE2^WT^, IE2^D184A^, and IE2^L173A^ proteins were generated by cell-free *in vitro* transcription/translation and subsequently incubated with increasing concentrations of GrB or GrM. Consistent with immunoblotting data (Fig 5A and 5B), IE2^WT^ was cleaved by GrB and GrM with concomitant appearance of multiple cleavage products (Fig 5C and 5D). In contrast, treatment of IE2^D184A^ with GrB showed only two detectable IE2 cleavage fragments (Fig 5C, right panel), and only one cleavage product remained detectable when IE2^L173A^ was treated with GrM (Fig 5D, right panel). Sequence alignment of different HCMV strains showed that amino acid regions surrounding IE2^D184^ and IE2^L173^ are highly conserved (Fig 5A and 5B, right panel). To investigate whether GrB or GrM-cleaved IE2 is still functionally active, IE2^1-184^ and IE2^185-579^ fragments that mimic GrB-cleaved IE2, and IE2^1-173^ and IE2^174-579^ fragments that mimic GrM-cleaved IE2 were constructed and expressed (Fig 5E and 5F). To examine the localization, HeLa cells were transfected with the various IE2 expression plasmids. As expected [25], full length IE2 localized in the nucleus (Fig 5G and 5H, upper row). IE2^1-184^ and IE2^1-173^ still localized in the nucleus (Fig 5G and 5H, middle row), whereas cytoplasmic localization was observed for IE2^185-579^ and IE2^174-579^ (Fig 5G and 5H, bottom row). IE2 is the critical transactivator of HCMV early genes [26]. As expected [26], full length IE2 efficiently activated HCMV UL112 early promoter as measured by a dual luciferase reporter assay (Fig 5I and 5J, left panel). In contrast, IE2^1-184^ or/and IE2^185-579^, and IE2^1-173^ or/and IE2^174-579^ did not activate HCMV UL112 early promoter anymore (Fig 5I and 5J, left panel). Immunoblot analysis confirmed that IE2 and its fragments were expressed (Fig 5K and 5L). Next, we investigated the competition between IE2 full length and its fragments in activating the HCMV UL112 early promoter. IE2^1-184^ and IE2^1-173^ did not compete with IE2 full length, whereas IE2^185-579^, IE2^174-579^, and relevant combinations caused dramatically reduction of residual HCMV UL112 early promoter activation (Fig 5I and 5J, right panel). Finally, to investigate whether cleavage of full-length IE2 by NK cells leads to IE2 functional disruption, we co-transfected IE2 and the HCMV UL112 early promoter reporter construct in HEK293T cells. Indeed, IE2-induced HCMV UL112 early promoter activation was inhibited by NK cells (Fig 5M). Taken together, GrB and GrM cleave IE2 at least after Asp^184^ and Leu^173^, respectively, resulting in IE2 aberrant cellular localization and functional abolishment of IE2 to transactivate the HCMV UL112 early promoter.

## Discussion

Current dogma is that cytotoxic lymphocytes secrete IFN-γ in the microenvironment to impair virus replication, followed by PFN-mediated delivery of granzymes inside target cells to trigger apoptosis and elimination of HCMV-infected cells [9, 12, 13, 20]. In the present study, we demonstrate that CD8^+^ T cells and NK cells can also deliver granzymes inside infected cells to control HCMV replication in a noncytotoxic manner by degrading essential HCMV proteins IE1 and IE2 (Figs 1 and 2), which are the initial key players during lytic HCMV replication. All human granzymes can directly cleave IE1 and/or IE2 (Fig 3), leading to functional disruption of these IE proteins (Figs 4 and 5).

The HCMV major immediate-early gene products IE1 and IE2 are nuclear phosphoproteins that play key roles in initiating lytic virus replication pathways [8, 27, 28]. IE2 is the major transactivator of HCMV early gene promoters and indispensable for HCMV replication [26]. This is supported by the observation that IE2-deficient HCMV fully loses the capacity of infectiousness and fails to express early genes [26]. Besides two nuclear localization signals (*i*.*e*., residues 145–151 and 321–328), IE2 contains activator domains (*i*.*e*., residues 25–85 and 544–579) that are involved in providing surfaces for initiation of transcriptional events, and a DNA binding domain (*i*.*e*., residues 346–579) that allows recognition of promoter elements [25, 29]. IE2 needs to translocate to the nucleus to fulfill its transactivation activity. GrB and GrM cleave IE2 after Asp^184^ and Leu^173^, generating short N-terminal and long C-terminal IE2 fragments (Fig 5). We find that short fragments IE2^1-173^ or IE2^1-184^ still localize in the nucleus, whereas long fragments IE2^174-579^ or IE2^185-579^ localize in both nucleus and cytoplasm (Fig 5). None of these IE2 fragments is functionally active in the transactivation of the UL112 early gene promoter (Fig 5), likely because granzymes separate and destroy functional IE2 activator and DNA binding domains to prevent IE2 from binding to the early promoters. In addition, long fragments IE2^174-579^ and IE2^185-579^ efficiently blocked the full-length IE2-induced HCMV UL112 early promoter activation (Fig 5), indicating these long fragments may compete with full-length IE2 binding to early promoter elements, resulting in a negative feedback loop to further reduce HCMV early promoter activation upon IE2 proteolysis by granzymes. This is consistent with the observation that NK cells inhibit IE2-induced HCMV UL112 early promoter activation in target cells (Fig 5). These findings strongly suggest that intracellularly delivered granzymes in infected cells abolish IE2-mediated transcriptional activation of viral early genes and subsequently inhibit HCMV replication.

Human granzymes are highly specific serine proteases [9, 13, 15–17]. Each granzyme cleaves after a unique and restricted order of amino acids (*i*.*e*., primary substrate specificity) and granzymes display only minor overlapping macromolecular substrate specificity [14, 16, 17, 22, 30]. Therefore, it is intriguing that all five human granzymes can cleave the same proteins (IE1 and/or IE2) at multiple distinct cleavage sites (Fig 3). Apparently, the immune system has created extreme redundancy to hit IE proteins. Moreover, we have previously demonstrated that GrM cleaves and inactivates the HCMV tegument protein pp71 [7], which is essential for IE mRNA expression [31]. GrM also targets an important host cell substrate (*i*.*e*., heterogeneous nuclear ribonucleoprotein K) that HCMV hijacks for its replication and that is necessary for efficient IE2 protein translation [32]. Hence, it seems conceivable that cytotoxic lymphocytes use granzymes to redundantly hit the initial IE machinery that HCMV requires for both lytic replication and reactivation from latency. To demonstrate that IE1/2 cleavage by granzymes released by killer cells plays an important role in HCMV immune control, a HCMV mutant should be generated that lacks all granzyme cleavage sites in both IE1 and IE2. However, making such a pan-granzyme resistant IE1/2 mutated virus is hampered by the large redundancy in (unidentified) granzyme cleavage sites and the likely detrimental consequences of manifold mutations on IE1/2 folding, structure, and function. Whether granzymes target additional viral proteins that play a role in the HCMV life cycle remains unknown.

Recently, Halle and coworkers have demonstrated by intravital imaging that CD8^+^ T cell-mediated killing of MCMV-infected cells *in vivo* is limited, and that single infected cells require multiple hits by CD8^+^ T cells to kill [18]. This proposed T cell cooperativity is compatible with our findings that CD8^+^ T cells and NK cells can control HCMV replication by killing of infected cells at high E:T ratios, whereas these killer cells leave HCMV-infected cells alive at lower E:T ratios (Fig 1). We further show that -at these low E:T ratios- cytotoxic cells can control HCMV replication, hence in a noncytotoxic manner (Fig 1). Noncytotoxic antiviral immune control may function as a failsafe mechanism to limit viral replication and spread to dampen HCMV replication during the time period to bridge optimal T cell cooperativity and killing of infected cells.

How killer cells determine the fate of HCMV-infected cells remains an intriguing question. This could be explained by the differential expression levels of granzyme (types) and PFN in individual CD8^+^ T cells and NK cells [33–36]. The fate of the infected cell may depend on the killing capacity of the respective cytotoxic cell. Alternatively, gradual accumulation of granzymes inside infected cells delivered by different single cytotoxic cells may ultimately turn the switch from noncytotoxic HCMV control (*e*.*g*., via cleavage of IE proteins) towards induction of apoptosis (*e*.*g*., via cleavage and activation of death substrates, like pro-caspases) [13, 16, 37, 38]. Finally, this tipping point could be manipulated by caspase inhibitors and other blockers of apoptosis that are synthesized by HCMV during productive infection [39–42]. Further studies are required to distinguish between these possibilities.

It is interesting to speculate that noncytotoxic targeting of IE proteins by killer cell granzymes, thus reducing the normal lytic function of these proteins, might have a role switching an infected cell into establishing a latent state. Alternatively, granzymes might help maintaining HCMV in a latent state by removing low levels of IE protein expression. This would be similar to latency-associated miR-UL112-1 that downregulates IE gene expression during latency [43]. Understanding how killer cells operate the noncytotoxic-cytotoxic switch and how this relates to lytic and latent antiviral immune control requires additional research.

## Materials and methods

### Ethics

Healthy adult CMV sero-positive (CMV+) and negative (CMV-) donors were recruited locally with ethical approval from the Addenbrookes National Health Service Hospital Trust institutional review board (Cambridge Research Ethics Committee); informed written consent was obtained from all volunteers in accordance with the Declaration of Helsinki (LREC 97/092).

### Cell culture, transfection, and cell-free protein lysates

Cells were cultured in a 5% CO_2_ atmosphere at 37°C. HFFs (ATCC #SCRC-1041), HeLa cells (ATCC #CCL-2), and human embryonic kidney (HEK293T, ATCC #CRL-11268) cells were maintained in Dulbecco’s modified Eagle medium (DMEM, Gibco) and NK cell line YT-Indy (Dept. of Pathology, University Medical Center Utrecht, Utrecht, The Netherlands) was cultured in RPMI 1640 medium (Gibco), supplemented with 10% fetal calf serum (FCS, BioWest), 100 μg/mL streptomycin and 100 units/mL penicillin (Invitrogen). Transfection was done using polyethylenimine (PEI, Polysciences Inc.). Cell-free protein lysates were generated by harvesting trypsinized cells, washing three times in ice-cold phosphate-buffered saline (PBS), and lysed by three cycles of freeze-thawing in liquid nitrogen. Lysates were centrifuged at 10,000 g for 10 min at 4°C. Cell-free supernatant was stored at -20°C until use.

### Virus, antibodies, and reagents

Clinical isolate UL32-GFP-Merlin-WT and GFP-Merlin delta US2-11 strains were kind gifts from Dr. R. Stanton (University of Cardiff, UK). Primary antibodies used for the following proteins were obtained from commercial sources: IE1/2 (mouse monoclonal, 11–003, Argene), GFP (mouse monoclonal, 7.1 and 13.1, Roche), pp65 (mouse monoclonal, 1-L-11, Santa Cruz), anti-ubiquitin (mouse monoclonal, clone Ubi-1, Merck). The antibody for HA (12CA5) is a mouse hybridoma supernatant. Secondary HRP-conjugated goat anti-mouse/rabbit antibodies were from Jackson ImmunoResearch Laboratories. Purified anti-human leukocyte antigen (HLA-A,B,C) antibody (clone W6/32), and isotype control antibody (clone MOPC-173) were from Biolegend, anti-IFN-γ antibody (clone AF-285-NA) was from R&D systems. Antibodies against IFN-α (Clone MMHA-6) and IFN-β (Clone MMHB-3) were from PBL Assay Science and isotype control antibody (MOPC-21) was from Biolegend. Alexa Fluor 488-conjugated goat anti-mouse was from Invitrogen. ZVAD-fmk was from ENZO Life Sciences. Instant Blue stain was from Expedeon and MG132 was obtained from Sigma. Immunoblotted proteins were detected using Enhanced Chemiluminescence (ECL) [44] or ECL prime western blotting detection reagents (GE Healthcare) and ChemiDoc XRS+ (Bio-Rad).

### Plasmids

pCGN-HA-IE1 and pCGN-HA-IE2 were generous gifts from Dr. T. Shenk (Princeton University, USA) [45]. pcDNA3.1-HA-IE1 and pcDNA3.1-HA-IE2 were generated by inserting the cDNAs of IE1 and IE2 extending from the BamHI to SalI sites of pCGN-HA-IE1 and the BamHI to XhoI sites of pCGN-HA-IE2, respectively, into the BamHI and XhoI sites of pcDNA3.1^+^ (Life Technologies) with Puro as selectable marker instead of Neo. pcDNA3.1-HA-IE1 and pcDNA3.1-HA-IE2 were used as templates to produce point mutants IE1^D398A^, IE1^L414A^ and IE2^D184A^, IE2^L173A^, respectively. pGloSensor-IE1, pGloSensor-IE2, and pGloSensor-IE1^L414A^ were constructed by inserting the IE1 and IE2 coding sequences extending from the AsiSI to PmeI sites of pcDNA3.1-HA-IE1, pcDNA3.1-HA-IE2, and pcDNA3.1-IE1^L414A^, respectively, into the AsiSI and PmeI sites of ProteaseGlo vector (Promega). While IE1^D398A^, IE2^D184A^, and IE2^L173A^ mutants in pGloSensor vector were produced by introducing point mutations in primers. pcDNA3.1-HA-IE1-FLAG-HA was produced by subcloning of a FLAG-HA PCR product into pcDNA3.1-HA-IE1. pcDNA3.1-IE1-FLAG-HA was produced by amplification of IE1-FLAG-HA using pcDNA3.1-HA-IE1-FLAG-HA as template and self-ligation. pcDNA3.1-HA-IE1-GFP was generated by a megaprimer approach with a H2B-GFP vector as a donor and pcDNA3.1-HA-IE1 as a recipient in a single PCR protocol as described previously [46]. To generate IE1 fragments with a C-terminal GFP tag that mimic GrB cleavage (pcDNA3.1-HA-IE1^1-398^-GFP and pcDNA3.1-HA-IE1^399-491^-GFP) or GrM cleavage (pcDNA3.1-HA-IE1^1-414^-GFP and pcDNA3.1-HA-IE1^415-491^-GFP), linear whole plasmid amplification was done by using Phusion polymerase (Thermo) not covering the part to be deleted and pcDNA3.1-HA-IE1-GFP as template. IE2 fragments that mimic GrB cleavage (pcDNA3.1-HA-IE2^1-184^ and pcDNA3.1-HA-IE2^185-579^) or GrM cleavage (pcDNA3.1-HA-IE2^1-173^ and pcDNA3.1-HA-IE2^174-579^) were produced in a similar way as IE1 fragments and pcDNA3.1-HA-IE2 as template. pGL4.24 10×IRE (Interferon Response Elements) was generated by insertion of tandem repeat of 5×IRE in pGL4.24 (Promega) between Eco53kI and EcoRV sites. 5×IRE was produced using forward primer 5’-GAGCTCTAGTTTCACTTTCCC-3’, reverse primer 5’-ATCCTCGAGGGGAAAGTG-3’ and GAGCTCTAGTTTCACTTTCCCTAGTTTCACTTTCCCTAGTTTCACTTTCCCTAGTTTCACTTTCCCTAGTTTCACTTTCCCCTCGAGGAT as template. The UL112 early promoter (EP) was obtained using forward primer 5’-ATAGATCTCCGCACAGAGGTAACAACGTG-3’, reverse primer 5’-ATAAGCTTGGCCGTGGAGCGAGTG-3’, and lysates of HCMV (AD169) infected HFFs as template, subcloned into pGL4.11 (Promega) by digestion with BglII and Hind III to generate pGL4.11-EP. Primers used for the other constructs are listed in S1 Table. All constructs were sequence verified.

### Generation of PFN-knockout NK cell line by CRISPR/Cas9

NK cell line YT-Indy was electroporated with Neon Transfection System (Thermo Fisher Scientific). Basic steps were performed as IDT manufacturer’s protocol described for delivery of ribonucleoprotein complexes into Jurkat T cells. Briefly, equal amounts of crRNA and tracrRNA were mixed before adding Cas9 to form the RNP complex. After 20 min incubation, cells were electroporated and grown in a 96-wells flat bottom plate. After 72 h, the knockout efficiency was checked by flow cytometry and PCR, subsequently verified by Sanger sequencing. Limiting dilution was used to generate single-cell clones of PFN-knockout NK cells that were confirmed by flow cytometry. Two crRNAs were combined for higher targeting efficiency. All steps were performed at room temperature. The PFN gRNA#1 target sequence was 5’-CACGGGGCAGGGACGGGCAG-3’. gRNA#2 target sequence was 5’-CGCAGCCACAAGTTCGTGCC-3’.

### HCMV dissemination assay

CMV serostatus was determined by HCMV specific IgG ELISA according to manufacturer’s instructions (Trinity Biotech). CD8^+^ T cells were obtained by positive selection from peripheral blood mononuclear cells (PBMCs), using anti-CD8 conjugated MicroBeads according to manufactures’ instructions (Miltenyi Biotec). The frequency of HCMV UL144, gB, pp65, IE1, IE2, US3, UL28 and pp71 specific CD8^+^ T cells was determined by FluoroSpot IFN-γ assay [47]. Autologous primary fibroblasts were established from dermal biopsies (University of Cambridge, UK). Autologous fibroblasts were seeded in 96- or 24-well plates and infected with GFP-HCMV (Merlin) at indicated multiplicity of infection (MOI) for 1 h. Fibroblasts were washed with PBS and CD8^+^ T cells from HCMV+/- donors were added at indicated E:T ratio in the presence or absence of anti-MHC-I, neutralizing IFN-γ, IFN-α and IFN-β antibodies for 9–14 days co-culture at 37°C. In parallel co-cultures, CD8^+^ T cells were washed off on day 10 and the HCMV infected fibroblasts were cultured until day 20. Fibroblasts were harvested, the percentage of GFP^+^ fibroblasts was measured by flow cytometry and viral genes mRNA level were analyzed by quantitative PCR (Q-PCR). For time course experiments, fibroblasts from 2 HCMV+ donors were infected with GFP-HCMV (Merlin, MOI = 0.05) and harvested at indicated time points. For E:T titration experiments, GFP-HCMV (Merlin, MOI = 0.08) infected-fibroblasts were co-cultured with autologous CD8^+^ T cells from 2 HCMV+ donors at indicated E:T ratios for 9 days. For NK cell experiments, GFP-HCMV (Merlin, MOI = 0.08) infected CMV+ donor-derived fibroblasts were co-cultured with non-autologous irradiated wide-type (WT) or PFN-KO NK cells (YT-Indy) at indicated E:T ratios for 9 days. For IE1/2 protein quantification, GFP-HCMV (Merlin, MOI = 0.8) infected HCMV+ donor-derived fibroblasts were co-cultured with autologous IE1-specific CD8^+^ T cells with increasing E:T ratios for 24 h, after which cells were washed and lysed by reducing Laemmli buffer and subjected to immunoblotting using antibodies against IE, pp65, or Hsp90β. Protein abundance was semi-quantified by Image Lab 5.1 software (Bio-Rad).

### Flow cytometry

Fibroblasts were harvested and fixed with 2% paraformaldehyde in PBS. The percentage of viral infection was analyzed via detection of GFP signal by FACS Calibur of LSRII Fortessa cytometer or Accuri (BD Biosciences) and data was analyzed with FlowJo 7.6 software (Tree Star Inc.) or BD CSampler software. The percentage of GFP^+^ fibroblasts was depicted as a proportion of the infected control.

### Q-PCR

Total RNA was extracted from fibroblasts using RNeasy Mini Kit (Qiagen) according to manufacturer’s protocol. DNA contamination was minimized using digestion with gDNA Wipeout reagent (Qiagen). cDNA synthesis was done using the Quantitect Reverse Transcription kit (Qiagen). Q-PCR was then carried out using a SYBR green-based reagent LUNA Universal Q-PCR Master Mix (NEB). Reactions were run on an ABi StepOnePlus using the following program: 95°C for 1 minute; then 45 cycles of 95°C for 15 seconds, 65°C 30 seconds. GAPDH was used as an internal control. Primers for HCMV genes and GAPDH are listed in S2 Table.

### LAK/NK cell-mediated cytotoxicity assay

HFFs were seeded in 96-wells plates and infected with GFP-HCMV (AD169) at a MOI of 0.4 for 2 h at 37°C. Cells were washed twice with serum-free DMEM and incubated for 24 h in supplemented DMEM. HeLa cells were transfected with pcDNA3.1-HA-IE1/2, pcDNA3.1-HA-IE1-FLAG-HA, pcDNA3.1-IE1-FLAG-HA, or empty vector combined with a GFP plasmid using PEI for 20 h. LAK cells were obtained by culturing PBMCs for 4 days in RPMI 1640 medium (Gibco) supplemented with 5% human AB serum, 2.5% sodium bicarbonate (Gibco) and 1000 units/ml of recombinant interleukin-2 (WOKA, Japan). Non-autologous LAK or NK cells (YT-Indy) were added at indicated E:T ratios for 6 h co-culture at 37°C. ZVAD-fmk was added prior to addition of effector cells. Cells were washed twice with PBS and directly lysed in reducing sample buffer for immunoblotting analysis.

### Production and purification of recombinant granzymes, IE1, and IE2

The cDNA encoding each human granzyme was amplified and cloned into yeast expression vector pPIC9 (Invitrogen). Catalytically inactive control granzymes (SA) were generated by mutation of Ser^195^ residue in the catalytic center into an Ala by QuikChange site-directed mutagenesis (Stratagene). All granzymes were expressed and purified as described previously [48]. Briefly, *Pichia pastoris* GS115 cells were transformed with the pPIC9-granzyme expression plasmids and expressed for 72 h in conditioned media, according to the manufacturers’ protocol (Invitrogen). Granzymes were purified to homogeneity by ion-exchange chromatography using an SP-Sepharose column (GE Healthcare). Purified granzymes were dialyzed against 50 mM Tris (pH 7.4) and 150 mM NaCl, and stored at -80°C. Recombinant granzymes were pure (>98%) as determined by SDS-PAGE and granzymes were active as determined by using synthetic chromogenic and macromolecular substrates [49]. Recombinant IE1 and IE2 proteins were produced and purified, essentially using protocols as described previously [50]. Briefly, IE1 and IE2 were expressed in fusion with GST using *E*.*coli* strain BL21 (DE3). After purification of the fusion proteins by affinity chromatography using glutathione-Sepharose columns (GE Healthcare, Bio-Sciences AB, Uppsala, Sweden), the GST-tag was cleaved by addition of PreScission protease (GE Healthcare). Free GST and GST-tagged PreScission Protease were removed by glutathione Sepharose affinity chromatography. IE1 and IE2 were then further purified by gel filtration.

### Granzymes cleavage assay *in vitro*

Nuclear fractions of HCMV (AD169)-infected HFFs, cell-free protein lysates of IE1/2 expressed in HeLa cells, or purified recombinant IE1 and IE2 were incubated with indicated concentrations of purified human GrA, GrB, GrH, GrK, and GrM or corresponding SA mutants at 37°C. Proteins were separated by SDS-PAGE, followed by immunoblotting or Instant Blue (Expedeon) staining.

### *In vitro* transcription/translation

To produce IE1^WT^, IE1^D398A^, and IE1^L414A^, IE2^WT^, IE2^D184A^, and IE2^L173A^ in a cell-free transcription/translation system *in vitro*, TnT SP6 High-Yield Protein Expression System (Promega, L3260) was used for all constructs in pGloSensor vector backbone according to the manufacturer’s protocol. To label the produced proteins, FluoroTect^TM^ GreenLys *in vitro* Translation Labeling System (Promega, L5001) was added to the reaction. Produced proteins were diluted and incubated with increasing concentrations of GrB or GrM for 3 h at 37°C in the dark. Samples were separated by SDS-PAGE and visualized on a Typhoon 9410 scanner (GE healthcare).

### Immunofluorescence

HeLa cells were grown on 8-well Falcon Culture Slides (BD Biosciences) and transfected with pcDNA3.1-HA-IE1-GFP, pcDNA3.1-HA-IE1^1-398^-GFP, pcDNA3.1-HA-IE1^399-491^-GFP, pcDNA3.1-HA-IE1^1-414^-GFP, pcDNA3.1-HA-IE1^415-491^-GFP, pcDNA3.1-HA-IE2, pcDNA3.1-HA-IE2^1-184^, pcDNA3.1-HA-IE2^185-579^, pcDNA3.1-HA-IE2^1-173^, or pcDNA3.1-HA-IE2^174-579^, respectively. After 20 h, cells were washed three times with PBS and fixed with 4% paraformaldehyde for 10 min. For IE2 constructs, cells were permeabilized for 10 min in 0.5% Triton X-100 and blocked with 2% BSA for 20 min followed by antibody incubation with anti-HA for 1 h, and Alexa Fluor 488-conjugated goat anti-mouse for 1 h. Fluorescence was analyzed by confocal scanning microscopes (LSM700, Zeiss).

### Dual luciferase reporter assay

To measure IE1 function, HEK293T cells were seeded on 24-wells plates and transfected with 30 ng H2B-mCherry, 20 ng pGL4.74 hRL-TK (Promega), 100 ng pGL4.24 10×IRE, increasing amounts of pcDNA3.1-HA-IE1-GFP, pcDNA3.1-HA-IE1^1-398^-GFP, pcDNA3.1-HA-IE1^399-491^-GFP, or pcDNA3.1-HA-IE1^1-414^-GFP, pcDNA3.1-HA-IE1^415-491^-GFP, complemented with empty vector to a total of 400 ng DNA per well. 24 h post transfection, cells were incubated with 5 ng/ml IFN-β (Peprotech) for 6 h after which the medium was removed and cells were lysed with passive lysis buffer (PLB, Promega). To measure IE2 function, HeLa cells were seeded on 24-wells plates and transfected with 30 ng H2B-mCherry, 20 ng pGL4.74 hRL-TK, 100 ng pGL4.11-EP, increasing amounts of pcDNA3.1-HA-IE2, pcDNA3.1-HA-IE2^1-184^, pcDNA3.1-HA-IE2^185-579^, or pcDNA3.1-HA-IE2^1-173^, pcDNA3.1-HA-IE2^174-579^, complemented with empty vector to a total of 400 ng DNA per well. For competition assay, constant amounts of pcDNA3.1-HA-IE2 (50 ng) together with 30 ng H2B-mCherry, 20 ng pGL4.74 hRL-TK, 100 ng pGL4.11-EP, increasing amounts of pcDNA3.1-HA-IE2^1-184^, or/and pcDNA3.1-HA-IE2^185-579^, pcDNA3.1-HA-IE2^1-173^, or/and pcDNA3.1-HA-IE2^174-579^, complemented with empty vector up to 400 ng DNA were used. After 24 h, cells were lysed with PLB. To monitor full-length IE2 functionality in the presence of NK cells, HEK293T cells were seeded on 24-wells plates and transfected with 30 ng H2B-mCherry, 20 ng pGL4.74 hRL-TK, 100 ng pGL4.11-EP, with or without 10 ng pcDNA3.1-HA-IE2, complemented with empty vector to a total of 400 ng DNA per well. After 24 h transfection, cells were cultured with or without NK cells (YT-Indy) in the presence of ZVAD-fmk (100μM) for 23 h and lysed with PLB. pGL4.74 hRL-TK was used for normalization and allows adequate comparison between samples. Dual Luciferase Reporter Assay System (Promega) was used to determine renilla and firefly luciferase activities in a Veritas Microplate Luminometer (Turner Biosystems) according to the manufacturer’s protocol (Promega). Protein expression was verified by subjecting PLB lysates to immunoblotting with an anti-HA antibody.

### Statistical analysis

Statistical analysis was performed in GraphPad Prism using one-way ANOVA with Turkeys multiple comparisons test or Student t test. *P<0.05, **P<0.01, ****P<0.0001.

## Supporting information

S1 Table Primers used for plasmid construction. (DOCX)

S2 Table Q-PCR primers. (DOCX)
