# Supplementary material for: Killer cell proteases can target viral immediate-early proteins to control human cytomegalovirus infection in a noncytotoxic manner
Source: PLoS Pathog. 2020 Apr 13;16(4):e1008426. doi: 10.1371/journal.ppat.1008426 (PMC7179929; doi:10.1371/journal.ppat.1008426)
Supplement: S1 Table — (DOCX) [file ppat.1008426.s001.docx]

**S1_Table. Primers used for plasmid construction**

| Plasmids | Forward primer(5’to 3’) | Reverse primer(5’to 3’) |
| --- | --- | --- |
| pcDNA3.1-IE1 | TGGATCCGCCGCCACCATGGCTTC | GACTCGAGTTACTGGTCAGCCTTGCT |
| pcDNA3.1-IE2 | TGGATCCGCCGCCACCATGGCTTC | AGTCGACTTACTGAGACTTGTTCCTCAGG |
| pcDNA3.1/pGloSensor-IE1^D398A^ | GCTTCTCTGGTGTCACCCCC | AGAGGAGCTGGCACCAGC |
| pcDNA3.1-IE1^L414A^ | GCGTCCTCAGTAATTGTGGCTGAG | AGGGATAGTCGCGGGTACAG |
| pcDNA3.1/pGloSensor-IE2^D184A^ | GCCTTTACCATCCAGTACCGCAAC | GGGCTCGGGCTTGATGTC |
| pcDNA3.1/pGloSensor-IE2^L173A^ | GCCATCAAACAGGAAGACATCAAG | GGGCAGCATGATAGGCG |
| pGloSensor-IE1/IE2 /IE1^L414A^ | TATGCGATCGCCATGGCTTCTAGCTATCCT | AGTTTAAACGGGCCCTCTAGAC |
| pcDNA3.1-IE1^1-398^ | TAACTCGAGTCTAGAGGGCC | ATCAGAGGAGCTGGCAC |
| pcDNA3.1-IE1^399-491^ | TCTCTGGTGTCACCCCC | TAGAATTGGGTACCGAGCTC |
| pcDNA3.1-IE1^1-414^ | TAACTCGAGTCTAGAGGGCC | CAGAGGGATAGTCGCGG |
| pcDNA3.1-IE1^415-491^ | TCCTCAGTAATTGTGGCTGA | TAGAATTGGGTACCGAGCTC |
| pcDNA3.1-IE2^1-184^ | TAACTCGAGTCTAGAGGGCCCG | GTCGGGCTCGGGCTTG |
| pcDNA3.1-IE2^185-579^ | TTTACCATCCAGTACCGCAAC | TAGAATTGGGTACCGAGCTC |
| pcDNA3.1-IE2^1-173^ | TAACTCGAGTCTAGAGGGCC | GAGGGGCAGCATGATAG |
| pcDNA3.1-IE2^174-579^ | ATCAAACAGGAAGACATCAAGC | TAGAATTGGGTACCGAGCTC |
| pcDNA3.1-IE1-GFP | GTGACTAGAAGCAAGGCTGACCAGATGGTGAGCAAGGGCGAG | CGGGCCCTCTAGACTCGAGTTATTACTTGTACAGCTCGTCCATG |
| pcDNA3.1-IE1^1-398^-GFP | ATGGTGAGCAAGGGCGAG | ATCAGAGGAGCTGGCAC |
| pcDNA3.1-IE1^399-491^-GFP | TCTCTGGTGTCACCCCC | TAGAATTGGGTACCGAGCTC |
| pcDNA3.1-IE1^1-414^-GFP | ATGGTGAGCAAGGGCGAG | CAGAGGGATAGTCGCGG |
| pcDNA3.1-IE1^415-491^-GFP | TCCTCAGTAATTGTGGCTGA | TAGAATTGGGTACCGAGCTC |
