# Supplementary material for: Killer cell proteases can target viral immediate-early proteins to control human cytomegalovirus infection in a noncytotoxic manner
Source: PLoS Pathog. 2020 Apr 13;16(4):e1008426. doi: 10.1371/journal.ppat.1008426 (PMC7179929; doi:10.1371/journal.ppat.1008426)
Supplement: S2 Table — (DOCX) [file ppat.1008426.s002.docx]

**S2_Table. Q-PCR primers.**

| Genes | Forward (5’to 3’) | Reverse(5’to 3’) |
| --- | --- | --- |
| IE1 | GTCCTGACAGAACTCGTCAAA | TAAAGGCGCCAGTGAATTTTTCTTC |
| IE2 | ATTGGCCGAAGAATCCCTCAAA | GAGGATGTCACCGAGTTCTGTC |
| UL44^a^ | TACAACAGCGTGTCGTGCTCCG | GGCGTAAAAAACATGCGTATCAAC |
| pp28^a^ | TTCACAACGTCCACCCACC | GTGTCCCATTCCCGACTCG |
| US2 | AGCACACGAAAAACCGCATC | TGCGGAAGTCATACACGCAT |
| US3^b^ | ACCGTGGATATGGTGGACAT | AACAGCAGACCCCAATTGTC |
| US6 | ATCTGCATCTGTGCAGTCCC | TTCTCTCTCTGTCTCCGCGA |
| US11 | TACTCCGAAACATCGGGCAG | CGCGGGTAGTATGCCTGAAT |
| GAPDH^c^ | TGCACCACCAACTGCTTAGC | GGCATGGACTGTGGTCATGAG |

^a^See ref. 1.

^b^See ref. 2.

^c^See ref. 3.

References

1. Omoto S, Mocarski ES. Cytomegalovirus UL91 is essential for transcription of viral true late (gamma2) genes. J Virol. 2013;87(15): 8651-8664.

2. Cheung AKL, Huang Y, Kwok HY, Chen M, Chen Z. Latent human cytomegalovirus enhances HIV-1 infection in CD34(+) progenitor cells. Blood Adv. 2017;1(5): 306-318.

3. Vandesompele J, De Preter K, Pattyn F, Poppe B, Van Roy N, De Paepe A, et al. Accurate normalization of real-time quantitative RT-PCR data by geometric averaging of multiple internal control genes. Genome Biol. 2002;3(7): RESEARCH0034.
